# Subtoxic Levels of Apigenin Inhibit Expression and Secretion of VEGF by Uveal Melanoma Cells via Suppression of ERK1/2 and PI3K/Akt Pathways

**DOI:** 10.1155/2013/817674

**Published:** 2013-10-31

**Authors:** Shih-Chun Chao, Sheng-Chieh Huang, Dan-Ning Hu, Hung-Yu Lin

**Affiliations:** ^1^Department of Ophthalmology, Show Chwan Memorial Hospital, Changhua 500, Taiwan; ^2^Institute of Electrical and Computer Engineering, National Chiao Tung University, Hsinchu 30010, Taiwan; ^3^Tissue Culture Center, The New York Eye and Ear Infirmary, NY 10003, USA; ^4^Department of Optometry, Yuan Pei University, Hsinchu 30015, Taiwan

## Abstract

The effects of apigenin on the expression of VEGF in uveal melanoma cells have not been reported. We studied this effect and relevant signaling pathways in two human uveal melanoma cell lines (SP6.5 and C918). ELISA assay revealed that the constitutive secretion of VEGF by uveal melanoma cells was 21-fold higher than that in normal uveal melanocytes. Apigenin at subtoxic levels (1–5 **μ**M) significantly suppressed the secretion of VEGF in a dose- and time-dependent manner in melanoma cells. VEGF levels in the conditioned culture media from SP6.5 and C918 cell lines treated with 5 **μ**M apigenin for 24 h reduced to 29% and 21% of those in cells not treated with apigenin, respectively. RT-PCR analysis found that apigenin also decreased the expression of VEGF mRNA in melanoma cells. ELISA study of various signal pathways showed that apigenin significantly decreased phosphorylated Akt and ERK1/2 but increased phosphorylated JNK1/2 and p38 MAPK levels in melanoma cells. PI3K/Akt or ERK1/2 inhibitors significantly decreased, but JNK1/2 and p38 MAPK inhibitors did not influence the secretion of VEGF by melanoma cells, suggesting that apigenin suppresses the secretion of VEGF mainly through the inhibition of PI3K/Akt and ERK1/2 pathways.

## 1. Introduction

Uveal melanoma is the most common primary malignant intraocular tumor in adults in western countries [1]. This malignant tumor develops in one of the most capillary-rich tissues of the body and has a purely hematogenous dissemination. The mortality rate of uveal melanoma, is high because of the frequent occurrence of metastases, mainly in the liver. There is no efficient treatment for metastatic uveal melanoma and most of these patients died within 6 months after the metastasis [2, 3].

Angiogenesis is required for tumor growth and metastasis. VEGF or VEGF A is a vascular endothelial mitogen and stimulator of angiogenesis. VEGF plays a critical role in tumor angiogenesis and in the growth and metastasis of various cancers [4, 5]. It has been reported that uveal melanoma cells have a high constitutive expression and secretion of VEGF [6, 7]. A high serum VEGF level is associated with metastasis of uveal melanoma [8, 9]. Therefore, inhibiting the secretion of VEGF is becoming a target for uveal melanoma therapy [6].

Flavonoids are a family of polyphenolic compounds synthesized by plants with a similar structure and are divided into subclasses, including anthocyanidins, flavanols, flavanones, flavonols, flavones, and isoflavones. Epidemiological and case-control studies have suggested that high intake of various flavonoids from vegetables and fruits were inversely associated with risk of various cancers [10, 11]. Apigenin is a flavonoid belonging to the flavone subgroup and is present in various fruits, vegetables, herbs, and Chinese traditional medications [10, 11]. Apigenin inhibits the growth and invasion of various types of cancer in experimental animals and in vitro [10, 11]. Recently, it has been reported that apigenin also inhibits the secretion of VEGF in several types of malignant tumor cells [12–21].

The effects of apigenin on the expression and secretion of VEGF by uveal melanoma cells have not been reported. We have developed the methodology for isolation, cultivation, and study of normal human uveal melanocytes, established many uveal melanocyte cell lines, and collected several human uveal melanoma cell lines [22–25]. The purpose of this study was to compare the constitutive secretion of VEGF in uveal melanoma with normal uveal melanocytes, and to investigate the effects of apigenin on the expression and secretion of VEGF by uveal melanoma cells and the relevant signal pathways.

## 2. Materials and Method

### 2.1. Reagents

F-12 culture medium, Dulbecco's modified Eagle's medium (DMEM), fetal bovine serum (FBS), phosphate buffered saline (PBS), 0.05% trypsin-0.02% EDTA solution, and gentamicin were purchased from GIBCO (Grand Island, NY, USA). Apigenin, isobutylmethylxanthine, cholera toxin, dimethyl sulfoxide (DMSO), and 3-[4,5-dimethylthiazol-2-yl]-2,5-diphenyltetrazolium bromide (MTT) were purchased from Sigma (St. Louis, MO, USA). Basic fibroblast growth factor was purchased from PeproTech (Rocky Hill, NJ, USA).

### 2.2. Cell Culture

The constitutive levels of VEGF secretion were tested in three human uveal melanoma cell lines (MP17, SP6.5 and C918) and compared to those in three different human normal uveal melanocyte cell lines. M17 melanoma cell line (isolated from a primary choroidal melanoma patient), and primary cultures of human normal uveal melanocytes (all from the choroid) were established in the Tissue Culture Center of the New York Eye and Ear Infirmary as previously reported [22]. SP6.5 melanoma cell line was isolated from a primary choroidal melanoma patient and was provided by Dr. Guy Pelletier (Research Center of Immunology, Quebec, Canada) [26]. Melanoma cell line C918 was derived from a choroidal melanoma patient with liver metastasis at the University of Iowa and was provided by Dr. Robert Folberg (University of Illinois, Chicago) and Dr. Xiaoliang Leon Xu (Memorial Sloan Kettering Cancer Center, NY) [27]. Both M17 and SP.6.5 cell lines were originated from nonmetastatic uveal melanoma, and C918 is a metastatic and aggressive melanoma cell line [22, 26, 27]. Uveal melanoma cells were cultured in DMEM culture medium with 10% FBS, and uveal melanocytes were cultured in FIC medium with 10% FBS [22]. Cells were incubated at 37°C in a CO_2_ regulated incubator in a humidified 95% air/5% CO_2_ atmosphere. After cultures reached confluence, cells were detached with trypsin-EDTA solution and passaged. All tissues were obtained with premortem consent in accordance with the laws and regulations in place in the various jurisdictions.

### 2.3. Comparison of VEGF Secretion between Uveal Melanoma Cells and Normal Melanocytes

Early passages of three cell lines of cultured uveal melanocytes and three cell lines of uveal melanoma (M17, SP6.5 and C918) were plated into 24-well plates at a density of 1 × 10^5^ per well. After 24 h, the culture medium was withdrawn, washed with PBS, and replaced with serum-free culture medium. Conditioned media were collected 24 h later, centrifuged, and the supernatants were stored at −70°C until analysis. All experiments were performed in triplicate.

### 2.4. MTT Assay for Cell Viability

Uveal melanoma cells were seeded into 96-well plates at a density of 5 × 10^3^ cells per well. Apigenin (1.08 mg) was dissolved in 1 mL DMSO to make a stock solution of 2 mM. The cells in the control group were cultured in medium containing the same levels of DMSO as in the apigenin solution. After 48 h cultured with apigenin, MTT solution (1 mg/mL, 50 **μ**L) was added. After 4 h incubation, the medium and MTT were aspirated and 100 **μ**L of DMSO was added. Optical density of the plates was determined with a microplate reader (Multiskan MCC/340, Fisher Scientific, Pittsburgh, PA, USA) at 540 nm. The optical density in control (untreated) cells was taken as 100% viability. All tests were performed in three independent experiments.

### 2.5. VEGF Secretion in Uveal Melanoma Cells with Apigenin Stimulation

Uveal melanoma cells (SP6.5 and C918) were plated into 24-well plates at a density of 1 × 10^5^ per well. In the dose-effect study, after 24 h culture, the cultured medium was withdrawn, washed, and replaced with serum-free medium. Apigenin at different concentrations (0, 1.0, 2.0, and 5.0 **μ**M) was added to the media. After 24 h, conditioned media were collected and stored as described above. In the time-effect study, apigenin at 5 **μ**M was added into the culture medium. Conditioned media were collected at 6, 12, and 24 h later and stored as described above. Cultures without apigenin were used as the control. All tests were performed in three independent experiments.

### 2.6. Measurement of VEGF Levels

The amount of VEGF protein in the conditioned media was determined using the human VEGF (VEGF-A) Quantikine ELISA kit (R&D Systems, Minneapolis, MN, USA) according to the manufacturer's instructions. Optical density was read by using a microplate reader at 450 nm. The amount of VEGF (pg/mL) was calculated from a standard curve. The sensitivity of this kit was 5 pg/mL.

### 2.7. RNA Isolation and RT-PCR

Uveal melanoma cells (SP6.5) were plated into 6-well plates at a density of 5 × 10^5^. After 24 h, the culture medium was replaced with serum-free culture medium. Apigenin at different concentrations (0, 1.0, 2.0, and 5.0 *μ*M) was added to the media, and cells were collected at 6 h later. After the culture medium was withdrawn, the cultures were washed with cold PBS, and cells were harvested by scraping with a rubber policeman. Cells cultured without apigenin were used as negative controls. After microcentrifuging at 800 ×g for 5 min at 4°C, cell pellets were collected for mRNA extraction. Total RNA was isolated with the RNeasy PureLink RNA Mini Kit (Life Technology, Carlsbad, CA, USA), according to the manufacturer's instructions. The SuperScript first-strand synthesis system for RT-PCR kit (Invitrogen, Camarillo, CA, USA) was used to perform cDNA synthesis. The PCR primers for glyceraldehyde-3-phosphate dehydrogenase (GAPDH) were TGAACTGAPIGENINGCTCTCCACC, CTGATGTACCAGTTGGGGAA. VEGF primers were AGGGCAGAATCATCACGAAGT, AGGGTCTCGATTGGATGGCA. Both primers were obtained from Invitrogen. The first-strand cDNAs were synthesized from 0.5 *μ*g of total RNA at 50°C for 50 min. PCR amplification was conducted in a GeneAmp PCR system 9700 (Applied Biosystems, Foster City, CA, USA) using the following parameters: first denaturation at 94°C for 5 min followed by 35 cycles of reactions of denaturation at 94°C for 30 s, annealing at 58°C for 45 s, extension at 72°C for 45 s, and last extension on for 5 min at 72°C. After amplification, samples were run on a 1% agarose gel (Invitrogen) in TBE (0.01 M Tris-borate) 0.001 M EDTA (Invitrogen) containing 2.0 **μ**g/mL ethidium bromide (Invitrogen). Bands were visualized and photographed on a UV transilluminator (ChemiDoc XRS System, Bio-Rad, Hercules, CA, USA).

### 2.8. Phosphorylated Akt, ERK, JNK, and p38 MAPK Assay

Uveal melanoma cells (SP6.5) were seeded into 6-well plates at a density of 1 × 10^6^. After 24 h, apigenin at different concentrations (0, 1.0, 2.0, and 5.0 **μ**M) was added. After 1 h, the cultures were washed with cold PBS, and cells were harvested by scraping with a rubber policeman. Cells cultured without apigenin were used as the negative controls. After microcentrifuging for 5 min at 4°C, pellets were treated with ice-cold Cell Extraction Buffer (Biosource, Carlsbad, CA, USA) with Protease Inhibitor Cocktail (Sigma) and PMSF (Biosource) for 30 min, with subsequent vortexing at 10 min intervals. Cell extractions were microcentrifuged at 13,000 rpm for 10 min at 4°C. The supernatants were collected and stored at −70°C until analysis. Phosphorylated Akt, extracellular signal-regulated kinases 1/2 (ERK1/2), c-Jun N-terminal kinase (JNK1/2), and p38 mitogen-activated protein kinase (MAPK) measurement were performed in triplicate by using phosphorylated Akt, ERK1/2, JNK1/2 and p38 MAPK ELISA kits (Biosource), respectively, according to the protocol outlined by the manufacturer, and were expressed as percentages of the control (cells not exposed to apigenin). The sensitivity of ERK, JNK, and p38 MAPK ELISA kits was 0.8 U/mL and was 1.6 U/mL for Akt ELISA kit.

### 2.9. Effects of Akt, ERK, JNK and p38 MAPK Inhibitors on Secretion of VEGF by Uveal Melanoma Cells

Uveal melanoma cells were plated into 24-well plates at a density of 1 × 10^5^ cells per well. After 24 h incubation, the medium was changed, and various signal inhibitors were added to the medium separately, including 10 **μ**M of LY294002 (PI3K/Akt inhibitor), UO1026 (ERK inhibitor), SP600125 (JNK inhibitor), and SB203580 (p38 MAPK inhibitor), all from Calbiochem, San Diego, CA, USA. Thirty min later, apigenin was added to the medium at a final concentration of 5 **μ**M. Cells cultured without any signal inhibitor were used as the controls. After 24 h incubation, the conditioned media were collected and stored. The VEGF protein levels in the supernatants were determined using the human VEGF Quantikine ELISA kit as described above. Tests were performed in triplicate.

### 2.10. Statistical Analysis

Statistical significances of difference of means throughout this study were calculated by ANOVA one-way test in comparing data from more than two groups and Student's *t*-test in comparing data between two groups. The data was analyzed using SPSS statistical software (SPSS Inc., Chicago, IL, USA). A difference at *P* < 0.05 was considered to be statistically significant.

## 3. Results

### 3.1. Comparison of VEGF Secretion between Uveal Melanoma Cells and Uveal Melanocytes

**Figure 1:**
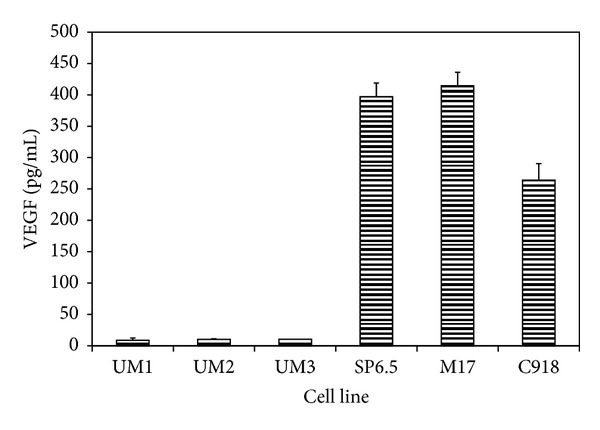
Constitutive secretion of VEGF by human uveal melanoma cells and normal uveal melanocytes. Three different primary cultures of uveal melanocytes (UM1-3) and uveal melanoma cells from 3 different cell lines (SP6.5, M17, and C918) were plated into 24-well plates. After 24 h, culture medium was removed and cultures were washed by PBS. Serum-free culture medium was added and cultured for 24 h. Conditioned media were collected, and the amount of VEGF protein was determined by using the human VEGF Quantikine ELISA kit. VEGF levels in conditioned culture medium were expressed as pg/mL. VEGF levels in culture medium from melanoma cells were significantly higher than that from normal melanocytes (*P* < 0.05). Data are mean ± SD (*n* = 3).

VEGF levels in the conditioned culture media from uveal melanoma and uveal melanocytes were measured and compared. In cells cultured with serum-free medium, VEGF levels were 359 ± 43.6 pg/mL and 14.3 ± 3.7 pg/mL in the conditioned media of uveal melanoma cells and uveal melanocytes, respectively (Figure 1). The VEGF levels in the conditioned medium from uveal melanoma cells were 25-fold those from normal uveal melanocytes; the difference was statistically significant (*P* < 0.05). The secretion of VEGF by uveal melanoma cells (1486 ± 243 pg/10^6^ cells/24 h) was also significantly greater than that by normal uveal melanocytes (67.4 ± 4.8 pg/10^6^ cells/24 h) (*P* < 0.05), indicating that uveal melanoma cells have a much higher constitutively secretion of VEGF (21-fold) as compared with their normal counterparts.

### 3.2. Effects of Apigenin at Different Levels on Cell Viability of Uveal Melanoma Cells

**Figure 2:**
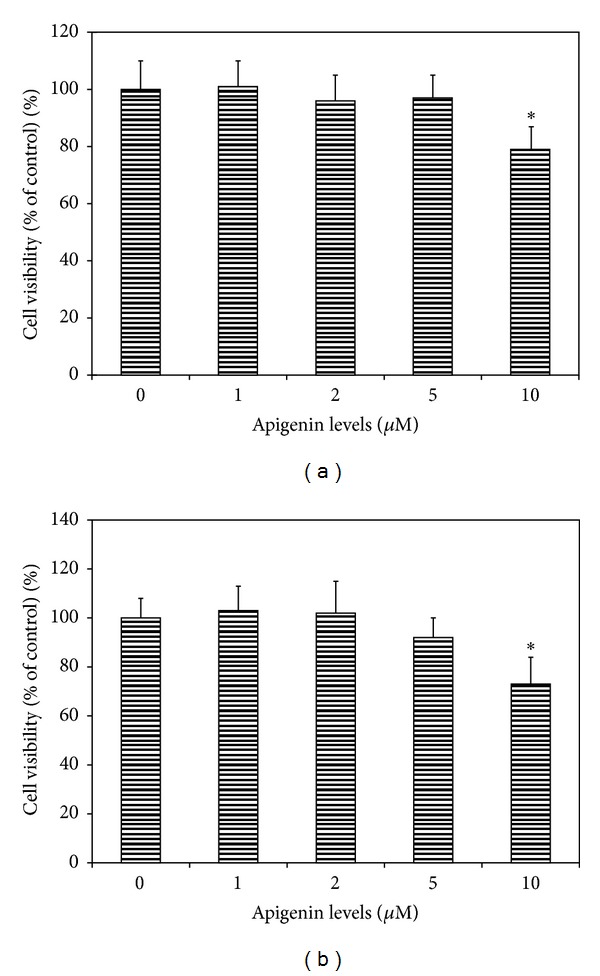
Dose effect of apigenin on cell viability of uveal melanoma cells. Human uveal melanoma cells SP6.5 (a) and C918 (b) were seeded into 96-well plates and treated with apigenin at various doses for 48 h, and cell viability was determined by MTT assay (see Methods). Apigenin at 1–5 **μ**M did not affect, whereas 10 **μ**M apigenin significantly decreased the cell viability of uveal melanoma cells. Data are mean ± SD (*n* = 3). *P < 0.05, versus control (cells cultured without apigenin).

In uveal melanoma cells (SP6.5 and C918) cultured with apigenin at 1.0–5.0 **μ**M, the reading of MTT test showed no significant difference as compared with cells cultured without apigenin (Figure 2), indicating that apigenin at 5 **μ**M or less did not affect the viability of both cell lines. Cell viability in uveal melanoma cells was decreased only at cells treated with 10 **μ**M apigenin (Figure 2). Therefore, apigenin at 5 **μ**M or less was used for the studies of effects of subtoxic levels of apigenin on the expression and secretion of VEGF from uveal melanoma cells.

### 3.3. Effects of Apigenin on VEGF Secretion by Uveal melanoma Cells

**Figure 3:**
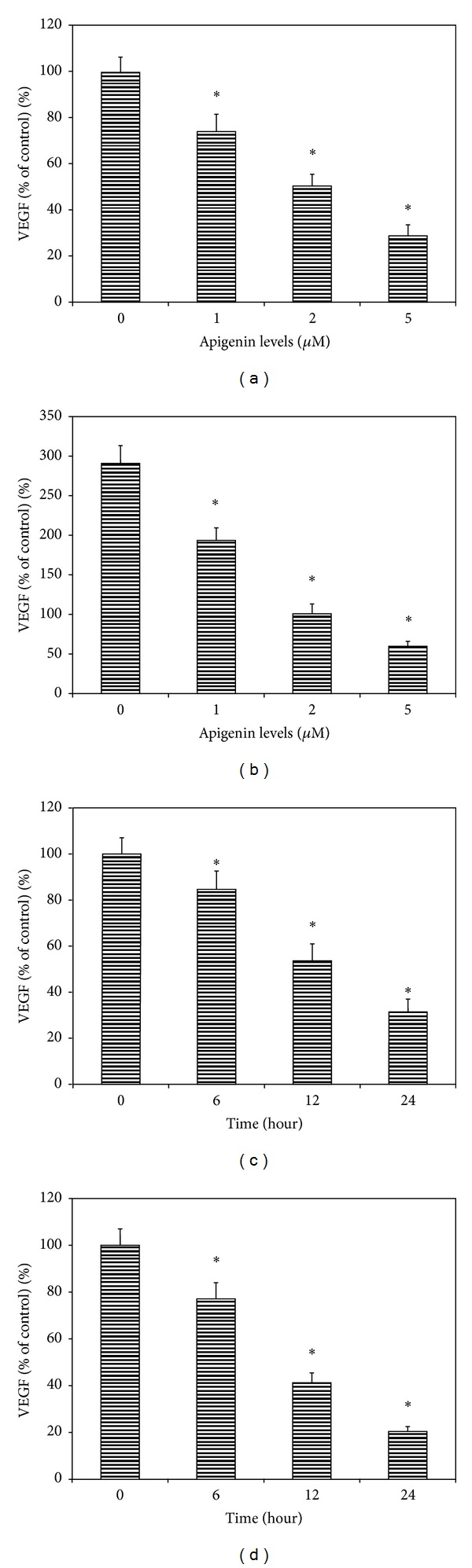
Dose- and time-effects of apigenin on the secretion of VEGF by human uveal melanoma cells. Cells were plated into 24-well plates. In the dose-effect study, apigenin at 0, 1, 2 and 5 **μ**M was added to the culture and incubated for 24 h, (a) SP6.5 cell line and (b) C918 cell line. In the time-effect study, apigenin at 5.0 **μ**M was added and conditioned medium was collected 6, 12 and 24 h later, (c) SP6.5 and (d) C918. The amount of VEGF protein in the conditioned medium was determined by using the human VEGF Quantikine ELISA kit. VEGF levels in the conditioned culture medium were expressed as the percentages of the controls. Apigenin significantly reduced the secretion of VEGF by uveal melanoma cells in a dose- and time-dependent manner. Data are mean ± SD (*n* = 3). *P < 0.05, versus control (cells cultured without apigenin).

Apigenin at different levels (1.0, 2.0, and 5.0 **μ**M) significantly decreased the VEGF protein levels in the conditioned medium in a dose-dependent manner (Figure 3). VEGF levels in the conditioned medium from cells (SP6.5) cultured without apigenin were 415 ± 29 pg/mL. VEGF levels in conditioned media from cells cultured with apigenin (1.0, 2.0, and 5.0 **μ**M) for 24 h were 74%, 51%, and 29% of the control values, respectively (Figure 3(a)). The difference of VEGF levels between apigenin treated cells and the controls was statistically significant at all levels of apigenin (*P* < 0.05). Studies in C918 melanoma cell line showed similar results (Figure 3(b)).

Apigenin inhibition of secretion of VEGF by uveal melanoma was also time dependent (Figure 3). VEGF levels in the conditioned media from SP6.5 melanoma cells cultured with apigenin (5.0 **μ**M) for 6, 12, and 24 h were 85%, 54%, and 31% of the control, respectively (Figure 3(c)). The difference of VEGF levels between apigenin treated cells and the controls was statistically significant in cells treated for 6, 12, and 24 h (*P* < 0.05). Studies in C918 melanoma cell line showed similar results (Figure 3(d)).

### 3.4. Effects of Apigenin on Expression of VEGF mRNA by Uveal Melanoma

**Figure 4:**
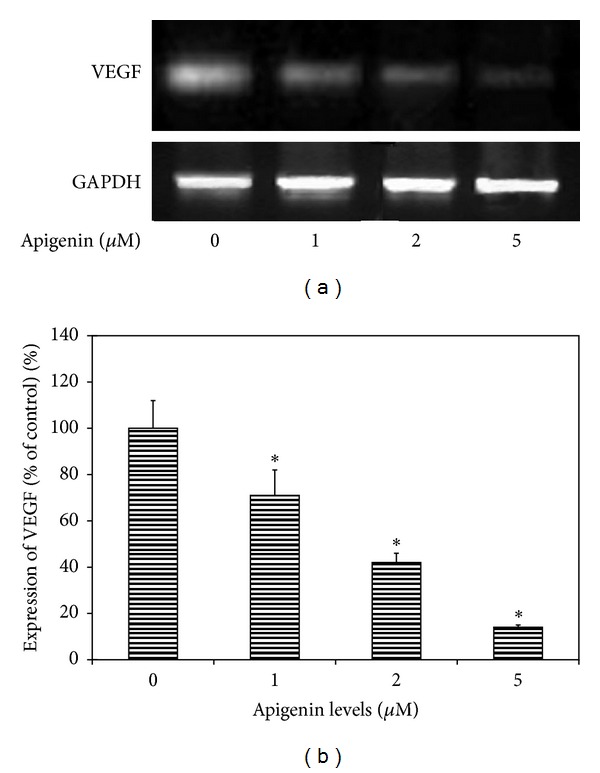
RT-PCR analysis for the effects of apigenin on the expression of VEGF mRNA in uveal melanoma cells. Representative RT-PCR profiles from three experiments showed the mRNA expressions of VEGF by uveal melanoma cells (SP6.5 cell line) exposed to apigenin at 0, 1, 2, and 5 **μ**M (a). After 6 h of culture with or without apigenin, cells were collected, mRNA was extracted, and RT-PCR analysis was performed as described in the text. GAPDH was used as an internal loading control. Apigenin inhibited the expression of VEGF mRNA in uveal melanoma cells in a dose-dependent manner (b).

The RT- PCR analysis demonstrated that VEGF mRNA was expressed in uveal melanoma cells (SP6.5 cell line) cultured without apigenin (Figure 4). Apigenin decreased VEGF mRNA expression in the uveal melanoma cells in a dose-dependent manner (Figure 4), indicating that apigenin also downregulates the expression of VEGF mRNA in uveal melanoma cells.

### 3.5. Effects of Apigenin on Phosphorylated Akt, ERK, JNK, and p38 MAPK Levels in Uveal Melanoma Cells

**Figure 5:**
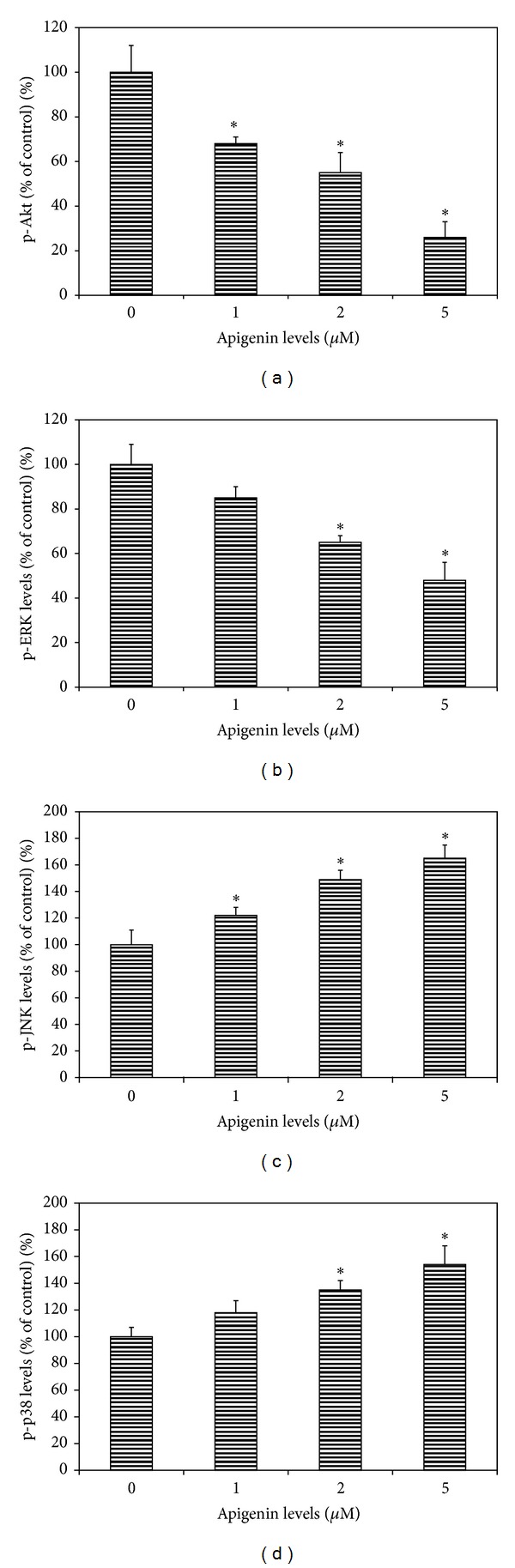
Dose effects of apigenin on phosphorylated Akt, ERK, JNK, and p38 MAPK levels in cultured uveal melanoma cells. Cells (SP6.5) were plated into 24-well plates. After 24 h incubation, apigenin at 0, 1, 2, and 5 **μ**M was added to the medium. Cells were collected 60 min later. The amount of phosphorylated Akt (p-Akt), ERK1/2 (p-ERK), JNK1/2 (p-JNK), and p38 MAPK (p-p38) in cell lysates was measured using the relevant ELISA kits and was expressed as the percentages of the controls (cells cultured without apigenin). Apigenin significantly decreased p-Akt (a) and p-ERK levels (b) and increased p-JNK (c) and p-p38 (d) levels in cell lysates in a dose-dependent manner. Data are mean ± SD (*n* = 3). *P < 0.05, versus control.

Apigenin treatment (5 **μ**M with 1 h incubation) significantly decreased both phosphorylated Akt and ERK1/2 levels in uveal melanoma cells (SP6.5 cell line) in a dose-dependent manner (Figures 5(a) and 5(b)). On the other hand, phosphorylated JNK1/2 and p38 MAPK levels were significantly increased in apigenin treated uveal melanoma cells in a dose-dependent manner (Figures 5(c) and 5(d)).

### 3.6. Effects of Various Signal Inhibitors on the Secretion of VEGF by Uveal Melanoma Cells

**Figure 6:**
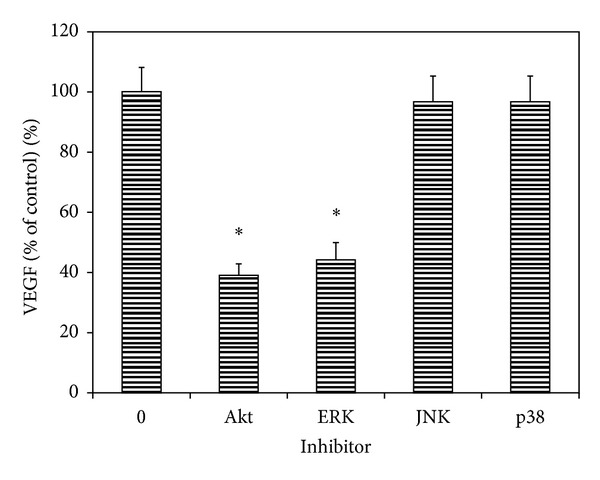
Effects of Akt and MAPK inhibitors on the constitutive secretion of VEGF by uveal melanoma cells. Cells were plated into 24-well plates. Various MAPK and Akt inhibitors were added to the medium separately, including LY294002 (PI3K/Akt inhibitor), UO1026 (ERK inhibitor), SP600125 (JNK inhibitor), and SB203580 (p38 MAPK inhibitor) at a final concentration of 10 **μ**M. After 24 h incubation, culture medium was collected, the VEGF levels were measured with human VEGF Quantikine ELISA kit and expressed as the percentages of the controls (cells cultured without any signal inhibitors). Akt and ERK inhibitors significantly decreased, whereas JNK and p38 inhibitors did not affect the VEGF levels in the conditioned culture medium from uveal melanoma cells. Data are mean ± SD (*n* = 3). *P < 0.05, versus control (cells cultured without inhibitor).

VEGF protein levels in the conditioned media from uveal melanoma cells (SP6.5 cell line) cultured without any signal inhibitors were 437 ± 36 pg/mL (control). Treatment of cells with LY294002 (PI3K/Akt inhibitor) and UO1026 (ERK inhibitor) significantly decreased VEGF levels in the conditioned media (*P* < 0.05) (Figure 6). VEGF levels in the conditioned media from SP600125 (JNK1/2 inhibitor) and SB203580 (p38 MAPK inhibitor) treated cells did not show significant changes as compared with the control (*P* > 0.05) (Figure 6). These results suggest that VEGF secretion by uveal melanoma cells is regulated by Akt and ERK1/2 and not by JNK1/2 and p38 MAPK signal pathways.

## 4. Discussion

Apigenin is a flavonoid belonging to the flavone structural class and chemically known as 4′, 5, 7,-trihydroxyflavone, with molecular formula C_15_H_10_O_5_. Apigenin is abundantly present in common fruits such as grapefruit, plant-derived beverages, and vegetables such as parsley, onions, oranges, tea, chamomile, and wheat sprouts. For centuries, apigenin has been utilized as a traditional or alternative medicine. One of the most common sources of apigenin consumed as single ingredient herbal tea is chamomile, prepared from the dried flowers of *Matricaria chamomilla* [10, 11]. Apigenin is present in various Chinese traditional medications, such as *Achillea millefolium, Apium graveolens, Buddleja officinalis, Cosmos bipinnata, Gingko biloba, Sabina chinensis, Taraxacum officinale, and Thymus serpyllum*, and have been used for the treatment of different diseases [28, 29]. In recent years, apigenin has been increasingly recognized as a cancer chemopreventive agent [10, 11].

Apigenin has gained particular interest in recent years as a beneficial and health promoting agent because of its low intrinsic toxicity and different effects in normal versus cancer cells [10, 11]. It has been reported that apigenin could inhibit the growth of cancers in experimental animal models [10, 11] In vitro studies suggested that apigenin induced apoptosis and inhibited the growth and invasion of various cultured cancer cells, including breast, cervical, colon, lung, ovarian, prostate, gastric, liver, and skin cancer cells [10, 11].

Angiogenesis is required for tumor growth and metastasis. VEGF is a critical regulator of tumor vascularization. The VEGF family consists of VEGF or VEGF-A, VEGF-B, VEGF-C, VEGF-D, VEGF-E (viral), and placenta growth factor [4]. VEGF family members signal by binding to members of a group of high-affinity receptors, VEGFR-1, -2, -3 and Neuropilin-1 and -2. VEGF-A binds to VEGFR-1, -2, and Neuropilin-1, and all of them are selectively though not exclusively expressed on vascular endothelium [4]. VEGF modulates tumor vascularization through its potent functions as a stimulator of endothelial cell survival, mitogenesis, migration, differentiation, and self-assembly [4, 5]. It is well established that VEGF is an important component of tumor growth, vascularity, and metastasis. VEGF expression, both in the tumor and in the circulation, is correlated with poor patient prognosis in several types of cancer [4, 5]. Cancer cells constitutively express VEGF proteins without apparent stimuli, which may provide a paracrine mechanism to induce angiogenesis [4, 5]. Therefore, using various drugs that inhibit the expression and secretion of VEGF by cancer cells is becoming a target for cancer therapy.

In uveal melanoma, it has been reported that VEGF mRNA and protein were detected in the tumor tissues [30–32]. VEGF levels were elevated in aqueous humor from uveal melanoma patients [32, 33]. Serum VEGF levels were elevated in uveal melanoma with metastasis [8, 9]. Uveal melanoma cell lines show a very high constitutive expression and secretion of VEGF [6, 7]. All of these results suggest that VEGF plays an important role in the growth, invasion, and metastasis of uveal melanoma.

It has been reported that apigenin suppressed the expression and secretion of VEGF in various cancer cells in vitro, including lung [21], prostate [13–15, 17], liver [18], and ovarian [12, 20] cancers. Apigenin also suppressed the VEGF expression and secretion in various experimental cancer models [16, 21].

The effects of apigenin on the expression of VEGF in uveal melanoma cells have not been reported. The present study found that uveal melanoma cells had a much higher constitutive secretion of VEGF as compared with normal uveal melanocytes, which is consistent with the previous report [7]. Apigenin suppressed the expression and secretion of VEGF in uveal melanoma cells in a dose-dependent manner, which is consistent with the inhibition of VEGF expression and secretion by apigenin in various types of cancer [12–21].

Constitutive activation of the ERK1/2 and PI3K/Akt pathways has been observed in many tumors and plays a very important role in tumor progression [4, 5]. Activation of both signal pathways leads to enhanced VEGF gene transcription [4, 5]. It has been reported that ERK and PI3K/Akt signal pathways are highly activated in uveal melanoma cells constitutively [34–37]. PI3K/Akt signal pathway in uveal melanoma cells also could be regulated by micro-RNA (*miRNA-34a*) [38].

In the present study, apigenin significantly inhibited the activation of ERK1/2 and PI3 K/Akt and increased the activation of JNK1/2 and p38 MAPK pathways in cultured uveal melanoma cells. However, only ERK1/2 and PI3 K/Akt inhibitors significantly reduced the secretion of VEGF by uveal melanoma cells, whereas JNK1/2 and p38 inhibitors did not influence the secretion of VEGF by melanoma cells in the present study. Therefore, it is most probably that apigenin suppresses the expression and secretion of VEGF through the inhibition of ERK1/2 and PI3 K/Akt pathways. This is consistent with the previous reports, which showed that the inhibition of ERK1/2 and/or PI3 K/Akt pathways by apigenin plays an important role in its inhibiting effects on VEGF expression in various malignant tumors [12–14, 17, 21].

## 5. Conclusion

 Apigenin at low and nontoxic levels significantly inhibits the expression and secretion of VEGF by uveal melanoma cells, suggesting that it is worth to study the use of apigenin as a chemopreventive and/or chemotherapeutic agent against uveal melanoma in the future.

## References

[1] Hu DN, Yu GP, McCormick SA, Schneider S, Finger PT (2005). Population-based incidence of uveal melanoma in various races and ethnic groups. *American Journal of Ophthalmology*.

[2] Kujala E, Mäkitie T, Kivelä T (2003). Very long-term prognosis of patients with malignant uveal melanoma. *Investigative Ophthalmology and Visual Science*.

[3] Augsburger JJ, Corrêa ZM, Shaikh AH (2009). Effectiveness of treatments for metastatic uveal melanoma. *American Journal of Ophthalmology*.

[4] Xie K, Wei D, Shi Q, Huang S (2004). Constitutive and inducible expression and regulation of vascular endothelial growth factor. *Cytokine and Growth Factor Reviews*.

[5] Loureiro RMB, D’Amore PA (2005). Transcriptional regulation of vascular endothelial growth factor in cancer. *Cytokine and Growth Factor Reviews*.

[6] Logan P, Burnier J, Burnier MN (2013). Vascular endothelial growth factor expression and inhibition in uveal melanoma cell lines. *Ecancermedicalscience*.

[7] Notting IC, Missotten GSOA, Sijmons B, Boonman ZFHM, Keunen JEE, van der Pluijm G (2006). Angiogenic profile of uveal melanoma. *Current Eye Research*.

[8] El Filali M, Missotten GSOA, Maat W (2010). Regulation of VEGF-A in uveal melanoma. *Investigative Ophthalmology and Visual Science*.

[9] Barak V, Pe’er J, Kalickman I, Frenkel S (2011). VEGF as a biomarker for metastatic uveal melanoma in humans. *Current Eye Research*.

[10] Shukla S, Gupta S (2010). Apigenin: a promising molecule for cancer prevention. *Pharmaceutical Research*.

[11] Patel D, Shukla S, Gupta S (2007). Apigenin and cancer chemoprevention: progress, potential and promise (review). *International Journal of Oncology*.

[12] Fang J, Xia C, Cao Z, Zheng JZ, Reed E, Jiang BH (2005). Apigenin inhibits VEGF and HIF-1 expression via PI3K/AKT/p70S6K1 and HDM2/p53 pathways. *FASEB Journal*.

[13] Fang J, Zhou Q, Liu LZ (2007). Apigenin inhibits tumor angiogenesis through decreasing HIF-1*α* and VEGF expression. *Carcinogenesis*.

[14] Mirzoeva S, Nam DK, Chiu K, Franzen CA, Bergan RC, Pelling JC (2008). Inhibition of HIF-1 alpha and VEGF expression by the chemopreventive bioflavonoid apigenin is accompanied by Akt inhibition in human prostate carcinoma PC3-M cells. *Molecular Carcinogenesis*.

[15] Shukla S, Gupta S (2004). Suppression of constitutive and tumor necrosis factor alpha-induced nuclear factor (NF)-kappaB activation and induction of apoptosis by apigenin in human prostate carcinoma PC-3 cells: correlation with down-regulation of NF-kappaB-responsive genes. *Clinical Cancer Research*.

[16] Mafuvadze B, Benakanakere I, López Pérez FR, Besch-Williford C, Ellersieck MR, Hyder SM (2011). Apigenin prevents development of medroxyprogesterone acetate-accelerated 7,12-dimethylbenz(a)anthracene-induced mammary tumors in sprague-dawley rats. *Cancer Prevention Research*.

[17] Shukla S, MacLennan GT, Fu P, Gupta S (2012). Apigenin attenuates insulin-like growth factor-I signaling in an autochthonous mouse prostate cancer model. *Pharmaceutical Research*.

[18] Kim BR, Jeon YK, Nam MJ (2011). A mechanism of apigenin-induced apoptosis is potentially related to anti-angiogenesis and anti-migration in human hepatocellular carcinoma cells. *Food and Chemical Toxicology*.

[19] Melstrom LG, Salabat MR, Ding XZ (2011). Apigenin down-regulates the hypoxia response genes: HIF-1*α*, GLUT-1, and VEGF in human pancreatic cancer cells. *Journal of Surgical Research*.

[20] Luo H, Jiang BH, King SM, Chen YC (2008). Inhibition of cell growth and VEGF expression in ovarian cancer cells by flavonoids. *Nutrition and Cancer*.

[21] Liu LZ, Fang J, Zhou Q, Hu X, Shi X, Jiang BH (2005). Apigenin inhibits expression of vascular endothelial growth factor and angiogenesis in human lung cancer cells: implication of chemoprevention of lung cancer. *Molecular Pharmacology*.

[22] Hu DN, McCormick SA, Ritch R, Pelton-Henrion K (1993). Studies of human uveal melanocytes in vitro: isolation, purification and cultivation of human uveal melanocytes. *Investigative Ophthalmology and Visual Science*.

[23] Hu DN (2000). Regulation of growth and melanogenesis of uveal melanocytes. *Pigment Cell Research*.

[24] Hu DN, McCormick SA, Woodward DF (2001). A functional study on prostanoid receptors involved in cultured human iridal melanocyte stimulation. *Experimental Eye Research*.

[25] Hu DN, Wakamatsu K, Ito S, McCormick SA (2009). Comparison of eumelanin and pheomelanin content between cultured uveal melanoma cells and normal uveal melanocytes. *Melanoma Research*.

[26] Soulieres D, Rousseau A, Deschenes J, Tremblay M, Tardif M, Pelletier G (1991). Characterization of gangliosides in human uveal melanoma cells. *International Journal of Cancer*.

[27] Daniels KJ, Boldt HC, Martin JA, Gardner LM, Meyer M, Folberg R (1996). Expression of type VI collagen in uveal melanoma: its role in pattern formation and tumor progression. *Laboratory Investigation*.

[28] Zhou JJ, Xie GR, Yan XJ (2004). *Phytochemistry of Chinese Traditional Medicine*.

[29] Shen YJ (2002). *Pharmacology of Chinese Traditional Medicine*.

[30] Boyd SR, Tan DSW, de Souza L (2002). Uveal melanomas express vascular endothelial growth factor and basic fibroblast growth factor and support endothelial cell growth. *British Journal of Ophthalmology*.

[31] Sheidow TG, Hooper PL, Crukley C, Young J, Heathcote JG (2000). Expression of vascular endothelial growth factor in uveal melanoma and its correlation with metastasis. *British Journal of Ophthalmology*.

[32] Missotten GSO, Notting IC, Schlingemann RO (2006). Vascular endothelial growth factor A in eyes with uveal melanoma. *Archives of Ophthalmology*.

[33] Boyd SR, Tan D, Bunce C (2002). Vascular endothelial growth factor is elevated in ocular fluids of eyes harbouring uveal melanoma: identification of a potential therapeutic window. *British Journal of Ophthalmology*.

[34] Calipel A, Mouriaux F, Glotin AL, Malecaze F, Faussat AM, Mascarelli F (2006). Extracellular signal-regulated kinase-dependent proliferation is mediated through the protein kinase A/B-Raf pathway in human uveal melanoma cells. *Journal of Biological Chemistry*.

[35] Zuidervaart W, van Nieuwpoort F, Stark M (2005). Activation of the MAPK pathway is a common event in uveal melanomas although it rarely occurs through mutation of BRAF or RAS. *British Journal of Cancer*.

[36] Pópulo H, Soares P, Rocha AS, Silva P, Lopes JM (2010). Evaluation of the mTOR pathway in ocular (uvea and conjunctiva) melanoma. *Melanoma Research*.

[37] Saraiva VS, Caissie AL, Segal L, Edelstein C, Burnier MN (2005). Immunohistochemical expression of phospho-Akt in uveal melanoma. *Melanoma Research*.

[38] Yan D, Zhou X, Chen X (2009). MicroRNA-34a inhibits uveal melanoma cell proliferation and migration through downregulation of c-Met. *Investigative Ophthalmology and Visual Science*.

